# Supplementary material for: Indispensable role of Galectin-3 in promoting quiescence of hematopoietic stem cells
Source: Nat Commun. 2021 Apr 9;12:2118. doi: 10.1038/s41467-021-22346-2 (PMC8035175; doi:10.1038/s41467-021-22346-2)
Supplement: Supplementary file 1 — Supplementary Information [file 41467_2021_22346_MOESM1_ESM.pdf]

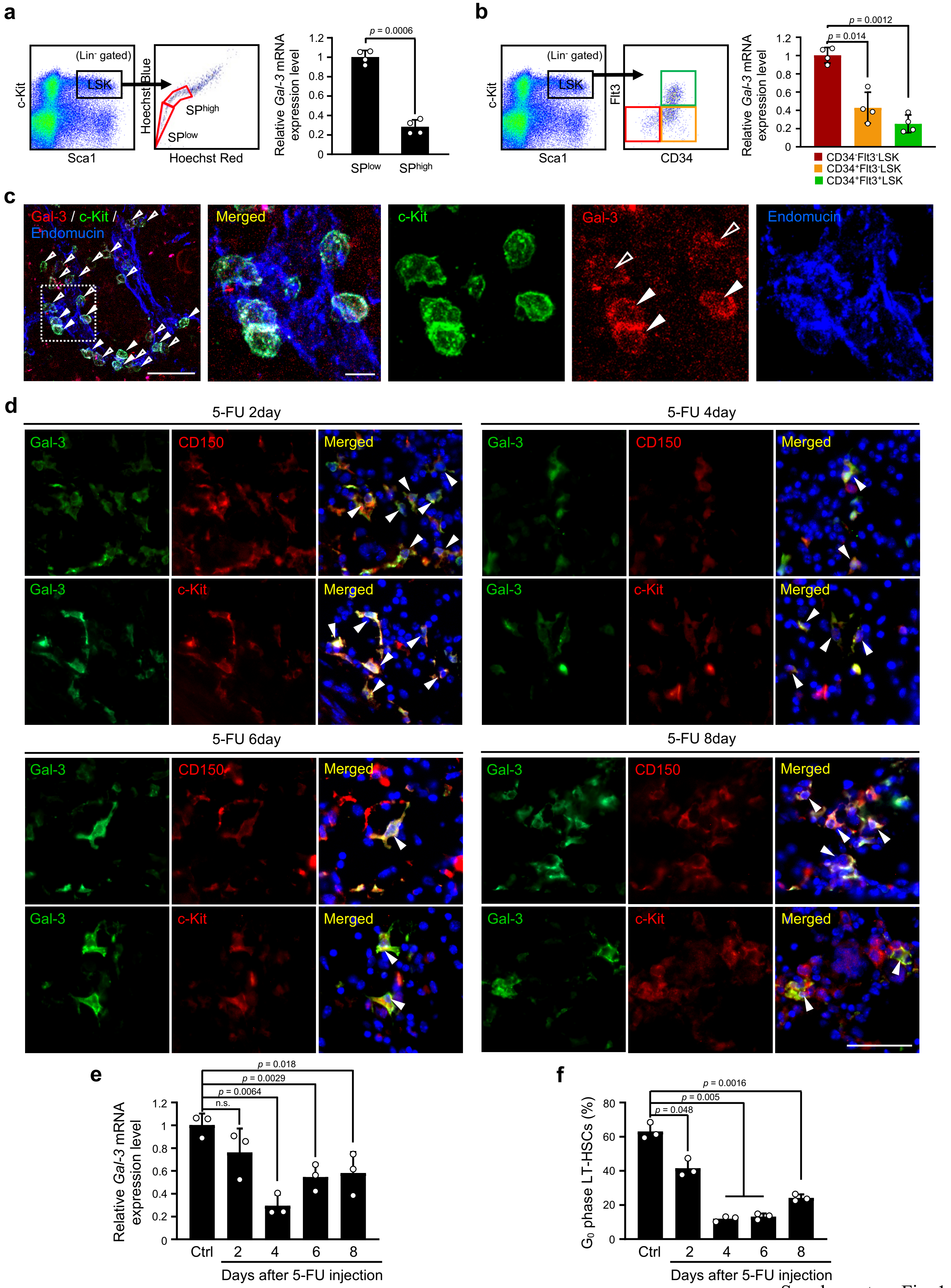


Supplementary Fig. 1

**Supplementary Fig. 1: High Gal-3 expression in HSCs is closely related to slow cell cycling or quiescence.**

(a) Relative expression level of *Gal-3* mRNA in BM-derived $SP^{low}$ and $SP^{high}$ LSK cells from WT mice (*n* = 4 biological replicates, compared by two-sided *t* test). Data are presented as mean values ± S.D..

(b) Relative expression level of *Gal-3* mRNA in $CD34^{-}Flt3^{-}$, $CD34^{+}Flt3^{-}$ and $CD34^{+}Flt3^{+}$ LSK cells from WT mice (*n* = 4 biological replicates, compared by two-sided *t* test). Data are presented as mean values ± S.D..

(c) Immunofluorescence staining of Gal-3 (red), c-Kit (green) and Endomucin (blue) in the BM sections from WT mice. Dashed box indicates areas shown at higher magnification in right panels. Transparent and white arrows indicate focal Gal-3-negative and Gal-3-positive cells. A representative image is shown (*n* = 3 biologically replicates). Scale bar, 50 μm and 10 μm (inset).

(d) Immunofluorescence staining of Gal-3 (green) and CD150 or c-Kit (red) in BM sections from WT mice at different time points after 5-FU treatment. DAPI (blue) was used to detect nuclei. Arrows indicate focal co-expression of Gal-3 and CD150 or c-Kit in the cells. A representative image is shown (*n* = 3 biologically replicates at each time point). Scale bar, 50 μm.

(e) Relative expression level of *Gal-3* mRNA in LT-HSCs ($CD150^{+}CD48^{-}Flt3^{-}LSK$) in BM of WT mice at different time points after 5-FU treatment. The control group (Ctrl) was treated with PBS (*n* = 3 biological replicates, compared by two-sided *t* test). Data are presented as mean values ± S.D. and n.s., not significant.

(f) The percentage of WT LT-HSCs ($CD150^{+}CD48^{-}Flt3^{-}LSK$) in $G_0$ phase at different time points after 5-FU treatment. The Ctrl was treated with PBS (*n* = 3 biological replicates, compared by two-sided *t* test). Data are presented as mean values ± S.D..

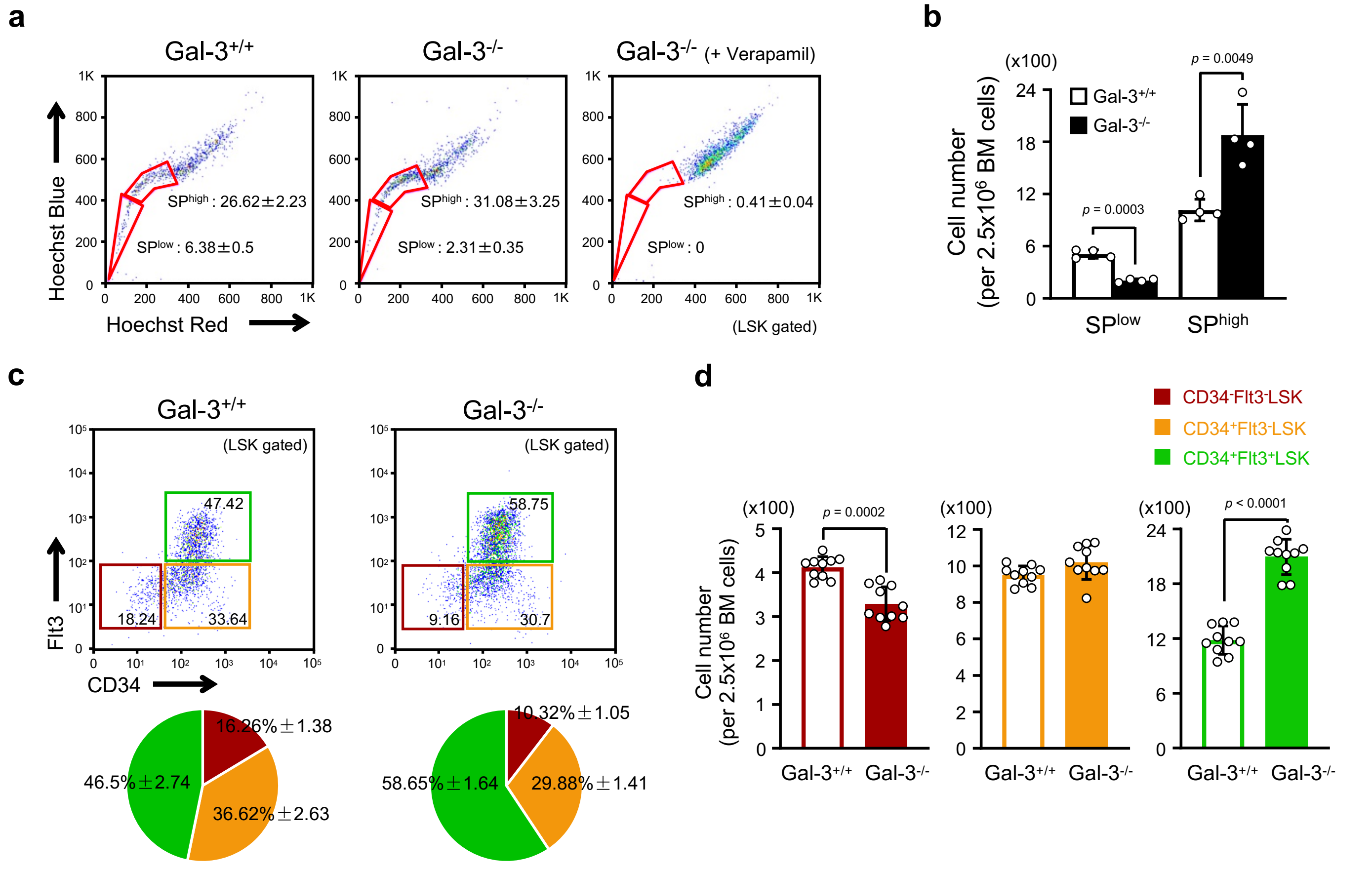


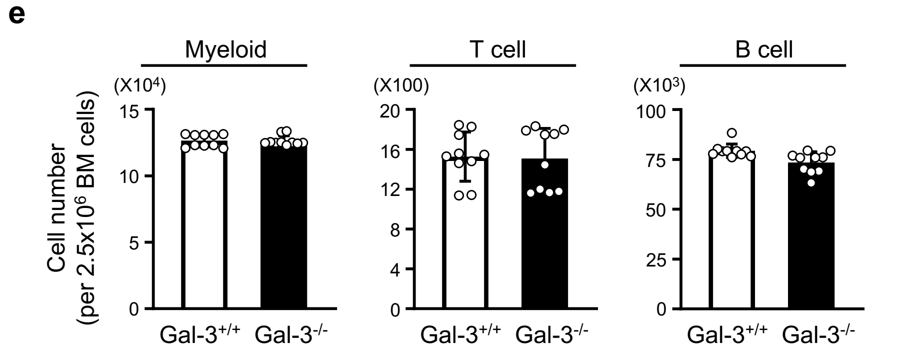


**f**

Measurement of PB parameters

| | Gal-3+/+ | Gal-3-/- | *p* Value |
|---|---|---|---|
| WBC (μl) | 2058 ± 1079 | 2016 ± 988 | n.s. |
| RBC (X10⁴/μl) | 746 ± 60 | 730 ± 78 | n.s. |
| Hb (g/dl) | 11 ± 0.88 | 12.2 ± 1.18 | n.s. |
| Ht (%) | 37.58 ± 3.27 | 38.68 ± 3.73 | n.s. |
| MCV (fl) | 50.3 ± 0.53 | 53.09 ± 1.50 | $p < 0.0001$ |
| MCH (pg) | 15.82 ± 0.28 | 16.74 ± 0.36 | $p < 0.0001$ |
| MCHC (%) | 31.46 ± 0.65 | 31.55 ± 0.64 | n.s. |
| PLT (X10⁴/μl) | 61.3 ± 15.39 | 68.84 ± 25.33 | n.s. |

**Supplementary Fig. 2: Gal-3 deficiency in LT-HSC does not alter the frequency of mature blood cells.**

(a) Representative flow cytometric analysis of $SP^{low}$ and $SP^{high}$ LSK populations in BM of Gal-$3^{+/+}$ or Gal-$3^{-/-}$ mice. The indicated values represented the percentage among LSK population, and disappearance of SP cells caused by Verapamil treatment (25 μg/ml). $n = 4$ biological replicates per genotype, compared by two-sided $t$ test. Data are presented as mean values ± S.D. and exact $p$ values: $SP^{low}$ : $p = 0.00035$ vs Gal-$3^{-/-}$; $SP^{high}$ : $p = 0.18$ vs Gal-$3^{-/-}$.

(b) Absolute number of $SP^{low}$ and $SP^{high}$ LSK cells in BM of Gal-$3^{+/+}$ or Gal-$3^{-/-}$ mice ($n = 4$ biological replicates per genotype, compared by two-sided $t$ test). Data are presented as mean values ± S.D..

(c) (Top) Representative flow cytometric analysis of $CD34^-Flt3^-$, $CD34^+Flt3^-$ and $CD34^+Flt3^+$ LSK populations in BM of Gal-$3^{+/+}$ or Gal-$3^{-/-}$ mice. (Bottom) Pie charts represent average frequencies of the 3 subsets within the LSK population ($n = 10$ biological replicates per genotype, compared by two-sided $t$ test). Data are presented as mean values ± S.D. and exact $p$ values: $CD34^-Flt3^-$LSK cells: $p < 0.0001$ vs Gal-$3^{-/-}$; $CD34^+Flt3^-$LSK cells: $p = 0.00018$ vs Gal-$3^{-/-}$; $CD34^+Flt3^+$ cells: $p < 0.0001$ vs Gal-$3^{-/-}$.

(d) Absolute number of $CD34^-Flt3^-$, $CD34^+Flt3^-$ and $CD34^+Flt3^+$ LSK cells in BM of Gal-$3^{+/+}$ or Gal-$3^{-/-}$ mice ($n = 10$ biological replicates per genotype, compared by two-sided $t$ test). Data are presented as mean values ± S.D..

(e) Absolute number of myeloid cells (Mac-1/Gr-$1^+$), T cells ($CD4/CD8^+$) and B cells ($B220^+$) in BM of Gal-$3^{+/+}$ or Gal-$3^{-/-}$ mice ($n = 10$ biological replicates per genotype).

(f) Comparison of blood cell counts in Gal-$3^{+/+}$ and Gal-$3^{-/-}$ mice ($n = 12$ biological replicates per genotype, compared by two-sided $t$ test). Data are presented as mean values ± S.D.. WBCs: white blood cells; RBCs: red blood cells; Hb: hemoglobin; Ht: hematocrit; MCV: mean corpuscular volume; MCH: mean corpuscular hemoglobin; MCHC: mean corpuscular hemoglobin concentration; PLTs: platelets. n.s., not significant.

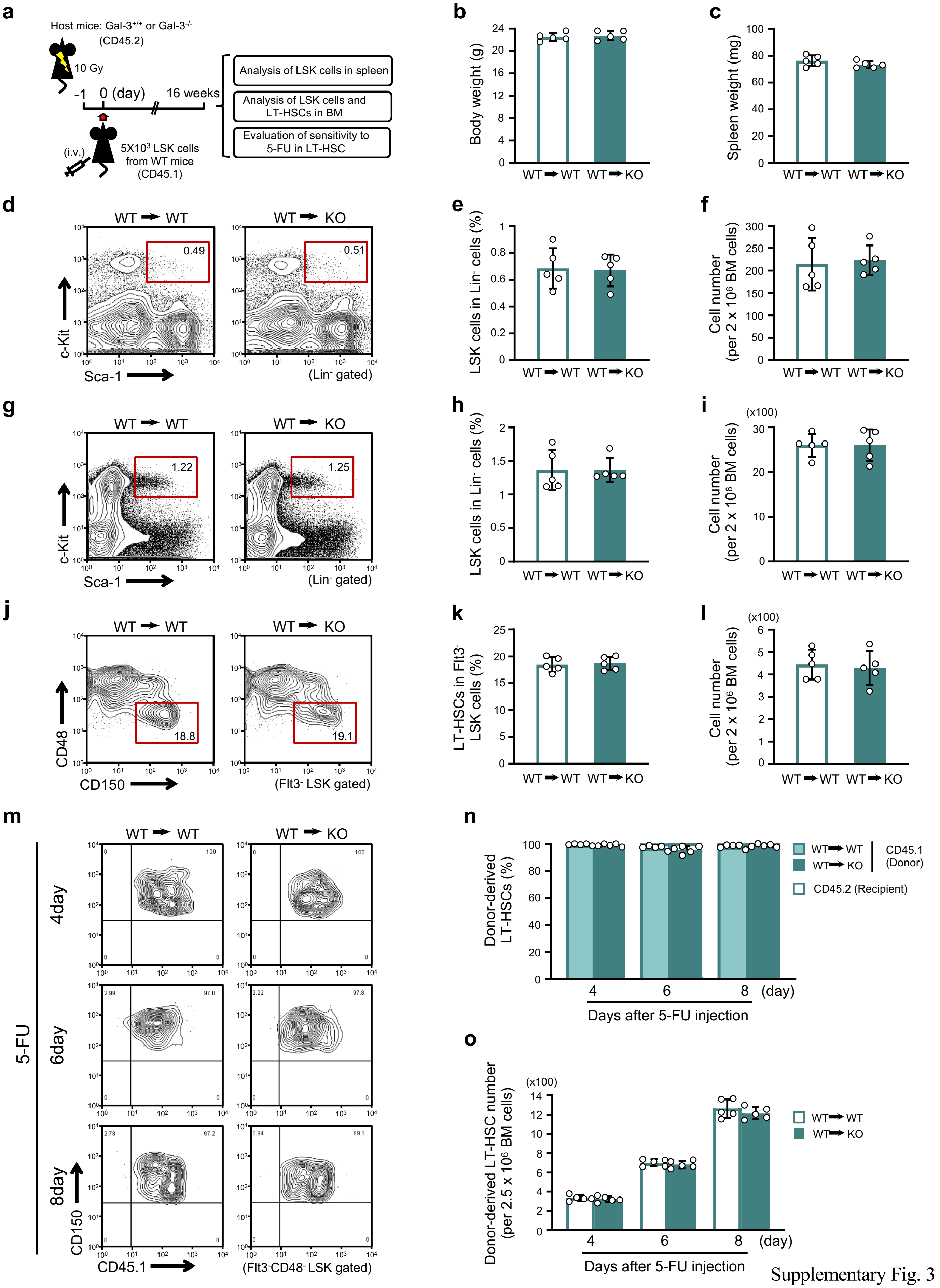


Supplementary Fig. 3

**Supplementary Fig. 3: Gal-3 deficiency in BM stromal cells does not affect HSC number and function.**

(a) Experimental schema for the BM-T chimeric mouse model. Donor (CD45.1)-derived WT LSK cells were transplanted into lethally-irradiated Gal-$3^{+/+}$ (WT) or Gal-$3^{-/-}$ (KO) mice (CD45.2). WT donors into WT recipients (WT→WT); WT donors into KO recipients (WT→KO).

(b) Weight of body after BM-T ($n = 5$ biological replicates per genotype).

(c) Weight of spleen after BM-T ($n = 5$ biological replicates per genotype).

(d) Representative flow cytometric analysis of donor-derived LSK cell frequencies (red box) in spleens of recipient mice (WT or KO).

(e) The percentage of donor-derived LSK cells in the $Lin^-$ population in spleens of recipient mice (WT or KO). $n = 5$ biological replicates per genotype.

(f) Absolute number of donor-derived LSK cells in spleens of recipient mice (WT or KO). $n = 5$ biological replicates per genotype.

(g) Representative flow cytometric analysis of donor-derived LSK cell frequencies (red box) in BM of recipient mice (WT or KO).

(h) The percentage of donor-derived LSK cells in the $Lin^-$ population in BM of recipient mice (WT or KO). $n = 5$ biological replicates per genotype.

(i) Absolute number of donor-derived LSK cells in BM of recipient mice (WT or KO). $n = 5$ biological replicates per genotype.

(j) Representative flow cytometric analysis of donor-derived LT-HSC ($CD150^+CD48^-Flt3^-LSK$) frequencies (red box) in BM of recipient mice (WT or KO).

(k) The percentage of donor-derived LT-HSCs ($CD150^+CD48^-Flt3^-LSK$) in the $Flt3^-LSK$ population in BM of recipient mice (WT or KO). $n = 5$ biological replicates per genotype.

(l) Absolute number of donor-derived LT-HSCs ($CD150^+CD48^-Flt3^-LSK$) in BM of recipient mice (WT or KO). $n = 5$ biological replicates per genotype.

(m) Representative flow cytometric analysis of donor-derived LT-HSC ($CD45.1^+CD150^+CD48^-Flt3^-LSK$) frequencies in BM of recipient mice (WT or KO) at different time points after 5-FU treatment.

(n) The percentage of donor-derived LT-HSCs ($CD45.1^+CD150^+CD48^-Flt3^-LSK$) in the $Flt3^-LSK$ population in BM of recipient mice (WT or KO). $n = 5$ biological replicates per genotype.

(o) Absolute number of donor-derived LT-HSCs ($CD45.1^+CD150^+CD48^-Flt3^-LSK$) in BM of recipient mice (WT or KO). $n = 5$ biological replicates per genotype.

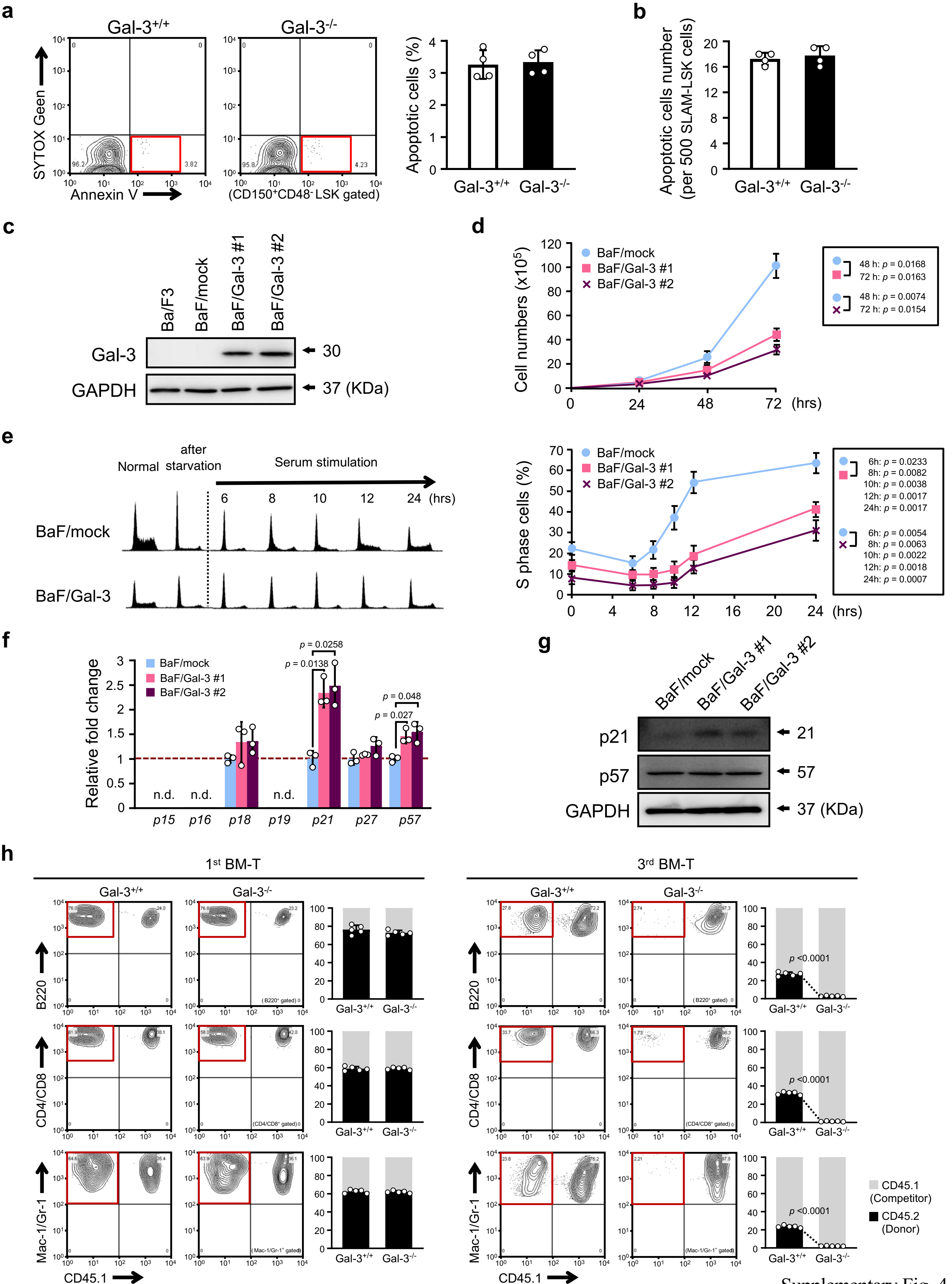


Supplementary Fig. 4

**Supplementary Fig. 4: Overexpression of Gal-3 suppresses cell-cycle progression in Ba/F3 cells.**

(a) (Left) Representative flow cytometric analysis of apoptotic Gal-$3^{+/+}$ or Gal-$3^{-/-}$ LT-HSCs ($CD150^{+}CD48^{-}$LSK, red box) using Annexin V and SYTOX Green staining. (Right) Bar graph showing the percentage of apoptotic cells (Annexin $V^{+}$/SYTOX $Green^{-}$) in LT-HSCs ($n = 4$ biological replicates per genotype).

(b) Absolute number of apoptotic cells in Gal-$3^{+/+}$ or Gal-$3^{-/-}$ LT-HSCs ($CD150^{+}CD48^{-}$LSK). $n = 4$ biological replicates per genotype.

(c) Western blotting of Gal-3 in Ba/F3, BaF/mock and BaF/Gal-3 cells (#1, #2).

(d) Growth curves of BaF/mock and BaF/Gal-3 cells (#1, #2). Cells ($1 \times 10^4$) were seeded in 6 well dishes and counted after 24, 48, and 72 hrs ($n = 3$ biological replicates, compared by two-sided $t$ test). Data are presented as mean values ± S.D..

(e) Cell cycle analysis of BaF/mock and BaF/Gal-3 cells (#1, #2) by staining with PI. (Left) Cells were harvested and cultured in under serum-free conditions for 12 hrs. After starvation, both were stimulated with serum and IL-3 for 24 hrs, and cell cycle time pints measured. (Right) Percentages of S phase cells were measured at each time point ($n = 3$ biological replicates, compared by two-sided $t$ test). Data are presented as mean values ± S.D..

(f) Relative expression level of mRNA for *p16* and *p21* family members in BaF/mock and BaF/Gal-3 cells (#1, #2) ($n = 3$ biological replicates, compared by two-sided $t$ test). Data are presented as mean values ± S.D. and n.d., not determined.

(g) Western blotting of p21 and p57 in BaF/mock and BaF/Gal-3 cells (#1, #2).

(h) Analysis of donor-derived B cells ($B220^{+}CD45.1^{-}$), T cells ($CD4/CD8^{+}CD45.1^{-}$) and myeloid (Mac-1/Gr-$1^{+}CD45.1^{-}$) in peripheral blood from primary and third BM-T recipients. Flow cytometric plots showed the relative contribution of mature blood cells from Gal-$3^{+/+}$ or Gal-$3^{-/-}$ donor-derived LT-HSCs (red boxes). Bar graph showing the percentage of donor- or competitor-derived B cells, T cells and myeloid cells in peripheral blood ($n = 5$ biological replicates per genotype, compared by two-sided $t$ test). Data are presented as mean values ± S.D..

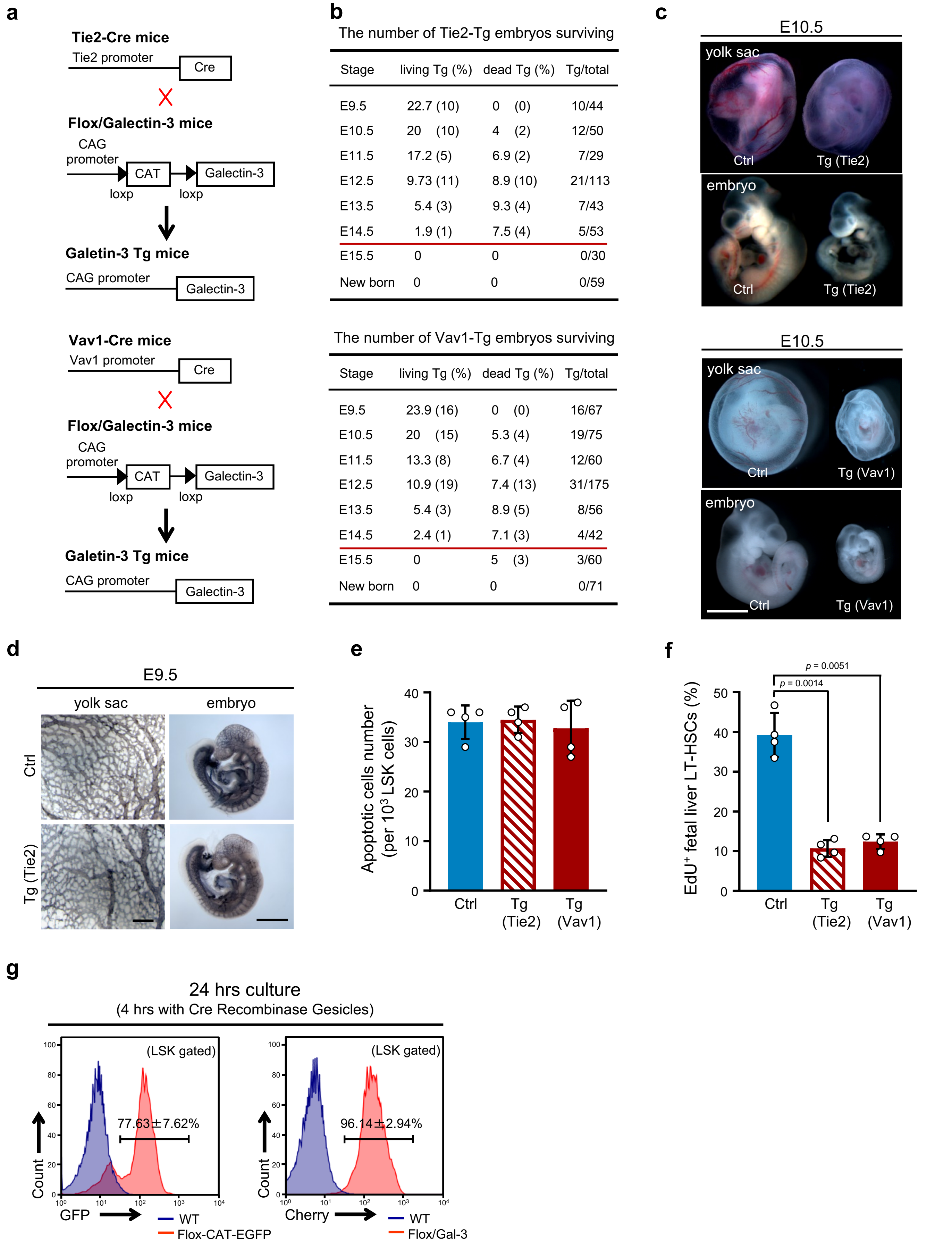


The number of Tie2-Tg embryos surviving

| Stage | living Tg (%) | dead Tg (%) | Tg/total |
|---|---|---|---|
| E9.5 | 22.7 (10) | 0 (0) | 10/44 |
| E10.5 | 20 (10) | 4 (2) | 12/50 |
| E11.5 | 17.2 (5) | 6.9 (2) | 7/29 |
| E12.5 | 9.73 (11) | 8.9 (10) | 21/113 |
| E13.5 | 5.4 (3) | 9.3 (4) | 7/43 |
| E14.5 | 1.9 (1) | 7.5 (4) | 5/53 |
| E15.5 | 0 | 0 | 0/30 |
| New born | 0 | 0 | 0/59 |

The number of Vav1-Tg embryos surviving

| Stage | living Tg (%) | dead Tg (%) | Tg/total |
|---|---|---|---|
| E9.5 | 23.9 (16) | 0 (0) | 16/67 |
| E10.5 | 20 (15) | 5.3 (4) | 19/75 |
| E11.5 | 13.3 (8) | 6.7 (4) | 12/60 |
| E12.5 | 10.9 (19) | 7.4 (13) | 31/175 |
| E13.5 | 5.4 (3) | 8.9 (5) | 8/56 |
| E14.5 | 2.4 (1) | 7.1 (3) | 4/42 |
| E15.5 | 0 | 5 (3) | 3/60 |
| New born | 0 | 0 | 0/71 |

Supplementary Fig. 5

**Supplementary Fig. 5: Early embryonic lethality in Gal-3 transgenic mice caused by anemia.**

(a) Schematic representation of the construct used to generate conditional transgenic mice. Overexpressed Gal-3 in Tie2-expressing cells or Vav-1-expressing cells. Black triangles in the middle figure indicate the loxp sites.

(b) Survival of Gal-3 Tg mice (Tie2-cre-Flox/Gal-3 or Vav-1-Cre-Flox/Gal-3) at different gestational ages. No Gal-3 Tg embryos survived beyond E15.5.

(c) Severe anemic phenotype displayed in E10.5 Gal-3 Tg embryos and yolk sac. Embryos were obtained by mating Tie2-Cre (Top) or Vav-1-Cre (Bottom) with Flox/Gal-3 (Ctrl) mice. A representative image is shown ($n = 5$ biological replicates per genotype). Scale bar, 1 mm.

(d) Whole mount staining of CD31 in the yolk sac and embryos of control (Ctrl, Flox/Gal-3) and Gal-3 Tg embryos (Tie2-Cre background) at E9.5. A representative image is shown ($n = 5$ biological replicates per genotype). Scale bar, 200 μm (yolk sac) and 1 mm (embryos).

(e) Apoptosis of LT-HSCs ($CD150^+CD48^-LSK$) from E12.5 Ctrl (Flox/Gal-3) and Gal-3 Tg fetal liver (Tie2-cre-Flox/Gal-3 or Vav-1-Cre-Flox/Gal-3) assessed by staining with Annexin V and SYTOX Green. Bar graph shows the absolute number of apoptotic cells in fetal liver-LT-HSCs ($n = 4$ biological replicates per genotype)

(f) Percentage of EdU-positive cells in fetal liver LT-HSCs ($CD150^+CD48^-LSK$). These cells were sorted from E12.5 Ctrl (Flox/Gal-3) and Gal-3 Tg embryos (Tie2-cre-Flox/Gal-3 or Vav-1-Cre-Flox/Gal-3) and labeled with EdU for 2 hrs ($n = 4$ biological replicates per genotype, compared by two-sided $t$ test). Data are presented as mean values ± S.D..

(g) Determination of Cre recombinase activity in ex vivo experiments. Purified LSK cells from the BM of Flox-CAT-EGFP or Flox/Gal-3 mice were treated with Cre Recombinase Gesicles for 4 hrs. One day later, fluorescent protein (GFP or mCherry) expression levels were estimated by flow cytometry.

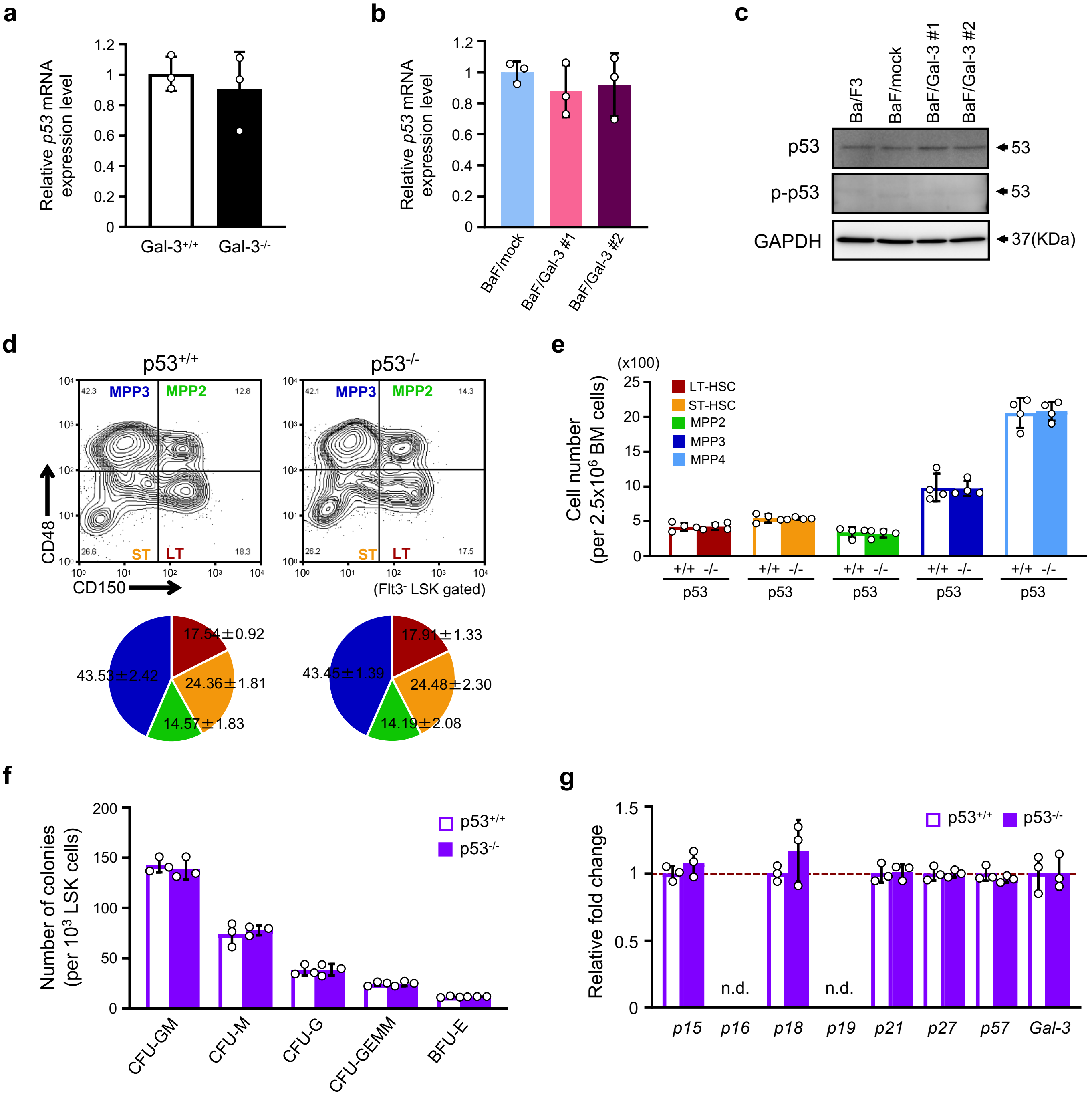


Supplementary Fig. 6

**Supplementary Fig. 6: Gal-3 regulates *p21* transcription in LT-HSC independent of p53.**

(a) Relative expression level of *p53* mRNA in LT-HSCs ($CD150^{+}CD48^{-}Flt3^{-}LSK$) in BM of Gal-$3^{+/+}$ or Gal-$3^{-/-}$ mice ($n = 3$ biological replicates per genotype).

(b) Relative expression level of *p53* mRNA in BaF/mock or BaF/Gal-3 cells (#1, #2). $n = 3$ biological replicates.

(c) Western blotting of p53 and p53 phosphorylation (p-p53) in BaF3, BaF/mock and BaF/Gal-3 cells (#1, #2).

(d) (Top) Representative flow cytometric analysis of LT-HSC, ST-HSC, MPP2 and MPP3 populations in BM of $p53^{+/+}$ or $p53^{-/-}$ mice. (Bottom) Pie charts represent average frequencies of the 4 subsets within the $Flt3^{-}$ LSK population ($n = 4$ biological replicates per genotype).

(e) Absolute number of LT-HSCs, ST-HSCs, MPP2, MPP3 and MPP4 in BM of $p53^{+/+}$ or $p53^{-/-}$ mice ($n = 4$ biological replicates per genotype).

(f) Colony forming potential of LSK cells from $p53^{+/+}$ or $p53^{-/-}$ mouse BM in CFU assays ($n = 3$ biological replicates per genotype).

(g) Relative expression level of mRNA for *p16* and *p21* family members in BM LT-HSCs ($CD150^{+}CD48^{-}Flt3^{-}LSK$) from $p53^{+/+}$ or $p53^{-/-}$ mice ($n = 3$ biological replicates per genotype). n.d., not determined.

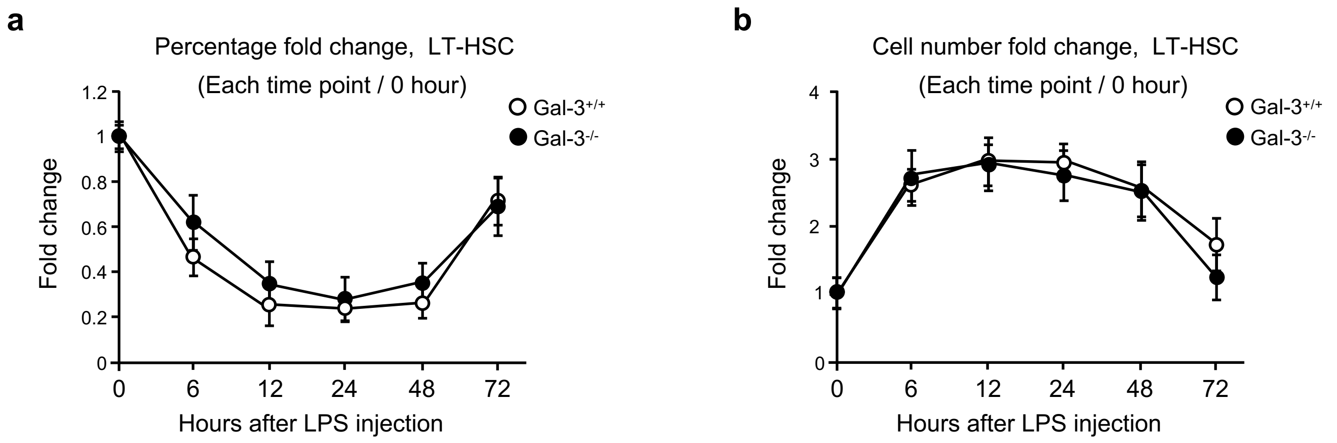


**Supplementary Fig. 7: Response of Gal-3 KO LT-HSCs to hematopoietic stress caused by LPS-treatment.**

(a) Fold-changes in percentages of LT-HSCs ($CD150^+CD48^-Flt3^-LSK$) in BM of Gal-$3^{+/+}$ or Gal-$3^{-/-}$ mice after LPS injection ($n = 3$ biological replicates per genotype at each time point).

(b) Fold-changes in numbers of LT-HSCs ($CD150^+CD48^-Flt3^-LSK$) in BM of Gal-$3^{+/+}$ or Gal-$3^{-/-}$ mice after LPS injection ($n = 3$ biological replicates per genotype at each time point).

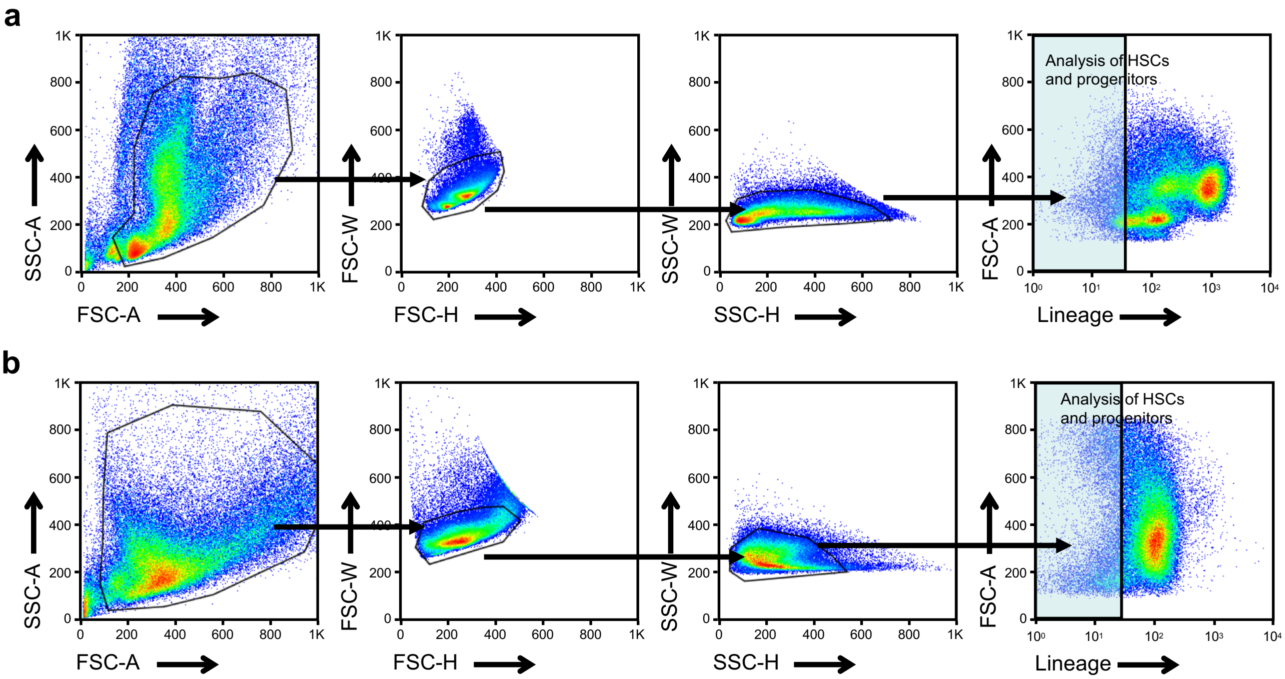


**Supplementary Fig. 8: Gating strategies used for cell analysis and sorting.**

(a) Gating strategy to analyze and BM cells from Gal-3$^{+/+}$, Gal-3$^{-/-}$ , Flox/Gal-3 and p53$^{-/-}$ mice (Fig.1 a-c, Fig.2 a-d, f, g, i, Fig.3 a-g, i, Fig.4 h-m, Fig.5 a-c, e-i, k, l, n, o, Fig.6 e, g, h, Supplementary Fig.1 a, b, e, f, Supplementary Fig.2 a-e, Supplementary Fig.3 d-o, Supplementary Fig.4 a, b, h, Supplementary Fig.5 g, Supplementary Fig.6 a, d-g, Supplementary Fig.7 a, b).

(b) Gating strategy to analyze and fetal liver cells from WT, control (Flox/Gal-3) and Gal-3 Tg (Tie2-Cre-Flox/Gal-3 or Vav-1-Cre-Flox/Gal-3) embryos (Fig.4 a-f, Supplementary Fig.5 e, f, ).

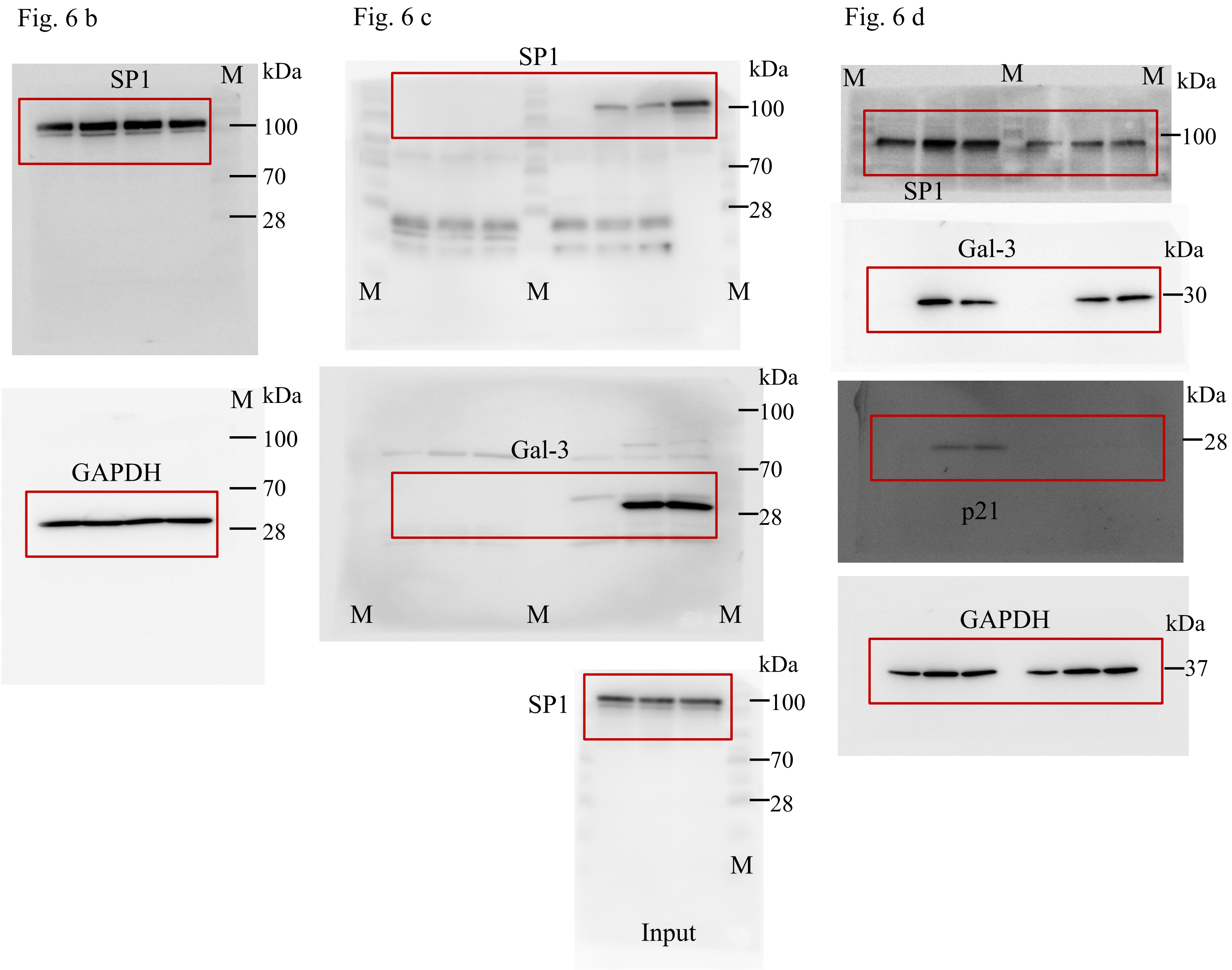


**Supplementary Fig. 9: Full scans of Western blots shown in main figures.**
Cropped areas are marked by red box. M: Marker

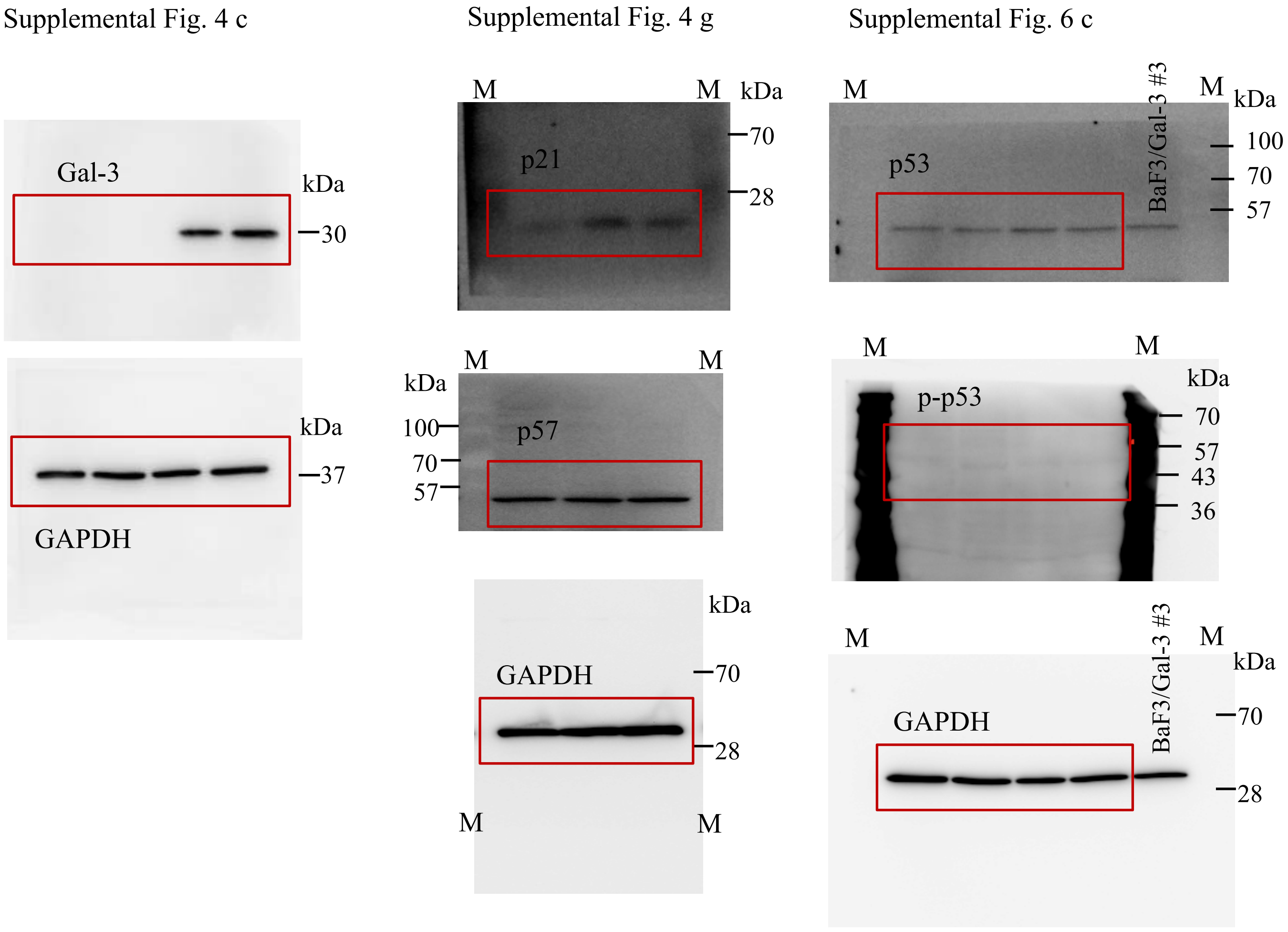


**Supplementary Fig. 10: Full scans of Western blots shown in supplementary figures.**
Cropped areas are marked by red box. M: Marker

**Supplementary Table 1**
Sequences of gene-specific primers for real-time PCR

| Gene name | Forward primer | Reverse primer |
|---|---|---|
| *Gal-3* | 5’-tgccctatgacctgccctt-3’ | 5’-tcctgcttcgtgttacacacaa-3’ |
| *p15* | 5’-agatcccaacgccctgaac-3’ | 5’-cccatcatcatgacctggatt-3’ |
| *p16* | 5’-cgtaccccgattcaggtgat-3’ | 5’-ttgagcagaagagctgctacgt-3’ |
| *p18* | 5’-tttcaaagaccgatgcgtatcc-3’ | 5’-ctatgtcagtatcagccagcaaa-3’ |
| *p19* | 5’-gccgcaccggaatcct-3’ | 5’-ttgagcagaagagctgctacgt-3’ |
| *p21* | 5’-taggggaattggagtcaggc-3’ | 5’-agagtgcaagacagcgacaa-3’ |
| *p27* | 5’-agatacgagtggcaggaggt-3’ | 5’-tcttaattcggagctgtttacgtc-3’ |
| *p53* | 5’-aaaggatgcccatgctacag-3’ | 5’-tatggcgggaagtagactgg-3’ |
| *p57* | 5’-accaatcagccagcagaaca-3’ | 5’-agttgaagtcccagcggttc-3’ |
| *GAPDH* | 5’-tggcaaagtggagattgttgcc-3’ | 5’-aagatggtgatgggcttcccg-3’ |

*p16* and *p21* family primer sequences were as previously described [Yamakawa, D. et al. Visualization of proliferative vascular endothelial cells in tumors in vivo by imaging their partner of sld5-1 promoter activity. *Am. J. Pathol.* 188, 1300-1314 (2018)].

**Supplementary Table 2**
Sequences of gene-specific primers for generation of different *p21* promoters

| Promoter fragment | Forward primer | Reverse primer |
|---|---|---|
| -302 to -1 | 5’-cccttgagagacaaggtgga-3’ | 5’-tttccccagacaaactgagc-3’ |
| -602 to -302 | 5’-ccaacataggcgggaattta-3’ | 5’-cttgcagtcctcactgatgc-3’ |
| -902 to -602 | 5’-gttgtcctcgccctcatcta-3’ | 5’-cacgcacgtacacagacaca-3’ |
| -1102 to -902 | 5’-agaggagcccgcactgtag-3’ | 5’-gttccgagtggaatggatgt-3’ |
| -1402 to -1102 | 5’-gcgaggaggtgactcattgt-3’ | 5’-atactgtgcccgccaaatag-3’ |
| -1702 to -1402 | 5’-gcttctggttgccaatgttt-3’ | 5’-aaacagggcacttggttcac-3’ |
| -2002 to -1702 | 5’-tgtgtgtggtgatgagtgga-3’ | 5’-tccaaggactggaagagtgg-3’ |
| -2302 to -2002 | 5’-aaggagtgggttggtcctg-3’ | 5’-tgtctggatatcgctgtgga-3’ |
| -2602 to -2302 | 5’-cccgaaacccaggattttat-3’ | 5’-ccacacctgggctattctct-3’ |
| -2902 to -2602 | 5’-caggctggtcttgaacctgt-3’ | 5’-aggcattcaaggtcgttttg-3’ |
