# Supplementary material for: Indispensable role of Galectin-3 in promoting quiescence of hematopoietic stem cells
Source: Nat Commun. 2021 Apr 9;12:2118. doi: 10.1038/s41467-021-22346-2 (PMC8035175; doi:10.1038/s41467-021-22346-2)
Supplement: Supplementary file 3 — Reporting Summary [file 41467_2021_22346_MOESM3_ESM.pdf]

nature research

Corresponding author(s): Nobuyuki Takakura

Last updated by author(s): Jan 25, 2021

Reporting Summary

Nature Research wishes to improve the reproducibility of the work that we publish. This form provides structure for consistency and transparency in reporting. For further information on Nature Research policies, see our Editorial Policies and the Editorial Policy Checklist.

Please do not complete any field with "not applicable" or n/a. Refer to the help text for what text to use if an item is not relevant to your study.
For final submission: please carefully check your responses for accuracy; you will not be able to make changes later.

Statistics

For all statistical analyses, confirm that the following items are present in the figure legend, table legend, main text, or Methods section.

n/a	Confirmed	
☐	☒	The exact sample size (*n*) for each experimental group/condition, given as a discrete number and unit of measurement
☐	☒	A statement on whether measurements were taken from distinct samples or whether the same sample was measured repeatedly
☐	☒	The statistical test(s) used AND whether they are one- or two-sided<br>*Only common tests should be described solely by name; describe more complex techniques in the Methods section.*
☒	☐	A description of all covariates tested
☒	☐	A description of any assumptions or corrections, such as tests of normality and adjustment for multiple comparisons
☒	☐	A full description of the statistical parameters including central tendency (e.g. means) or other basic estimates (e.g. regression coefficient) AND variation (e.g. standard deviation) or associated estimates of uncertainty (e.g. confidence intervals)
☒	☐	For null hypothesis testing, the test statistic (e.g. *F*, *t*, *r*) with confidence intervals, effect sizes, degrees of freedom and *P* value noted<br>*Give P values as exact values whenever suitable.*
☒	☐	For Bayesian analysis, information on the choice of priors and Markov chain Monte Carlo settings
☒	☐	For hierarchical and complex designs, identification of the appropriate level for tests and full reporting of outcomes
☒	☐	Estimates of effect sizes (e.g. Cohen's *d*, Pearson's *r*), indicating how they were calculated

Our web collection on statistics for biologists contains articles on many of the points above.

Software and code

Policy information about availability of computer code

Data collection	Flow cytometry: FACS Diva software (Version 1.5.1)<br>Quantitative real-time PCR: LightCycler 96 System (Version 1.1)<br>Western blotting: Amersham Imager 680 system (Version 2.0)
Data analysis	Flow cytometry: FlowJo (Version 10.7.1).<br>Quantitative real-time PCR: LightCycler 96 SW (Version 1.1).<br>Image processing: Photoshop CS6 (Version 13.0.1) and ImageJ (64-bit Java 1.8.0-172)<br>Quantification: Volocity (Version 6.3)<br>Statistical analysis: GraphPad Prism 9

For manuscripts utilizing custom algorithms or software that are central to the research but not yet described in published literature, software must be made available to editors and reviewers. We strongly encourage code deposition in a community repository (e.g. GitHub). See the Nature Research guidelines for submitting code & software for further information.

Data

Policy information about availability of data

All manuscripts must include a data availability statement. This statement should provide the following information, where applicable:

- Accession codes, unique identifiers, or web links for publicly available datasets
- A list of figures that have associated raw data
- A description of any restrictions on data availability

The authors declare that all data supporting the findings of this study are available within the article and its Supplementary information files. Source data for Figures 1–6 and Supplementary Figures. 1–10 are provided with the paper. Any other data are available from the authors upon reasonable request.

Field-specific reporting

Please select the one below that is the best fit for your research. If you are not sure, read the appropriate sections before making your selection.

☒ Life sciences ☐ Behavioural & social sciences ☐ Ecological, evolutionary & environmental sciences

For a reference copy of the document with all sections, see nature.com/documents/nr-reporting-summary-flat.pdf

Life sciences study design

All studies must disclose on these points even when the disclosure is negative.

Sample size	Sample size was determined based on the minimum number of animals / replicates that would be required to do power calculations. The number of independent samples analyses is provided for each experiment in the figure legend.<br>For immunohistochemical staining, femur or bone marrow cells samples collected from at least 3 independent wild-type (or mutant) litters were processed for immunohistochemical staining.<br>For 5-FU- or LPS-induced bone marrow cell death experimental model, competitive reconstitution assay, flow cytometric analysis sample size was determined based on the number of mutant mouse available in same age and similar weight.
Data exclusions	No data points were excluded.
Replication	For all data, 3 independent experiments were performed. We have used at least 3 biological replicates for each experiment and all attempts at replication were successful.
Randomization	Mice of the same age and similar weight were randomly assigned to experimental or control groups.
Blinding	Investigators were not blinded to allocation during cell and mouse assays. It was not possible to be blinded the mice due to the obvious morphological differences between wild-type and mutant (Tg).

Reporting for specific materials, systems and methods

We require information from authors about some types of materials, experimental systems and methods used in many studies. Here, indicate whether each material, system or method listed is relevant to your study. If you are not sure if a list item applies to your research, read the appropriate section before selecting a response.

Materials & experimental systems

n/a	Involved in the study	
☐	☒	Antibodies
☐	☒	Eukaryotic cell lines
☒	☐	Palaeontology and archaeology
☐	☒	Animals and other organisms
☒	☐	Human research participants
☒	☐	Clinical data
☒	☐	Dual use research of concern

Methods

n/a	Involved in the study	
☒	☐	ChIP-seq
☐	☒	Flow cytometry
☒	☐	MRI-based neuroimaging

Antibodies

Antibodies used	Conjugated antibodies:<br>Mouse Lineage Antibody Cocktail-Per CP Cy5.5 (BD Biosciences; Cat: 561317; Lot: 8300510), Gr-1-FITC (BD Biosciences; RB6-8C5; Cat: 11-5931-85; Lot: E00739-1632), Mac-1-FITC (BD Biosciences; M1/70; Cat: 553310; Lot: 3010776), B220-FITC (BD Biosciences; RA3-6B2; Cat: 11-0452-85, Lot: E00309-1631), CD4 (BD Biosciences; RM4-5; Cat: 11-0042-85, Lot: E00083-1633), CD8-FITC (BD Biosciences; 53-6.7; Cat: 11-0081-85; Lot: E00116-1634), CD34-Biotin (eBioscience; RAM34; Cat: 13-0341; Lot: 4313540), CD45-APC (eBioscience; 30-F11; Cat: 17-0451-82; Lot: 4291970), CD45.1-PE-Cy7 (BioLegend; A20; Cat: 110730; Lot: B168737), Flt3/CD135-APC (BioLegend; A2F10; Cat:135310; Lot: B234045), Sca-1-PE-Cy7 (BioLegend; E13-161.7; Cat: 122514; Lot: B280435), Sca-1-BV421 (BioLegend; D7; Cat: 108128; Lot: B266526), c-Kit-APC-Cy7 (BioLegend; 2B8; Cat: 105826; Lot: B280920), CD93-APC (BioLegend; AA4.1; Cat: 136510; Lot: B150985), CD150-PE (BioLegend; TC15-12F12.2; Cat: 115904; Lot: B270365), CD150-BV421 (BioLegend; TC15-12F12.2; Cat: 115926; Lot: B265580), CD150-Alexa 647 (BioLegend; TC15-12F12.2; Cat: 115918; Lot: B292223), CD48-FITC (BioLegend; HM48-1; Cat: 103404; Lot: B243598), CD48-APC (BioLegend; HM48-1; Cat: 103412; Lot: B181146), Endomucin-PE (eBioscience; V.7C7; Cat: 12-5851-82; Lot: 2207554). Biotinylated antibodies were visualized with PE-conjugated streptavidin (BD Biosciences; Cat: 554061; Lot: 3346899).<br><br>Unconjugated antibodies:<br>Gal-3/MAC-2 (Cedarlane; M3/38; Cat: CL8942AP; Lot: 1942223A), c-Kit/CD117 (R&D Systems; Cat: AF1356), Endomucin (eBioscience; V.7C7; Cat: 14-5851-82; Lot: 1939586), p21 (Abcam; Cat: ab109199; Lot: GR296632-16), p21 (eBiosciences; Cat: 14-6715; Lot: E05079-1634), p57 (Abcam; Cat: ab75974), phospho-AKT (Cell Signaling Technology; Ser413; Lot: 19), p53 (Cell Signaling Technology;

	1C12; Lot: 10), phospho-p53 (Cell Signaling Technology; Ser15; Lot: 19), Sp1 (Merck Millipore; Cat: 07-645; Lot: 2794880), GAPDH (6C5; Merck Millipore; Cat: MAB374; Lot: 2792998); p65 (Cell Signaling Technology; D14E12; Lot: 13), CD31 (BD Biosciences; MEC13.3; Cat: 553370; Lot: 7257819), Ki-67 (eBioscience; SolA15; Cat: 14-5698-82; Lot: 2056928). Horseradish-peroxidase-conjugated goat anti-mouse (Jackson Laboratories; Cat: 115-035-003; Lot: 125435), anti-rat (Jackson Laboratories; Cat: 112-035-003; Lot: 113737) and anti-rabbit IgG (Jackson Laboratories; Cat: 111-035-003; Lot: 115952).
Validation	All antibodies used in the study were obtained from commercial vendors. All antibodies were validated by their manufacturers for the application (western blotting, flow cytometry and immunohistochemistry) and species (mouse) used in this study, and were supported by publications. See manufacturers websites for validation statements, found in technical datasheet.<br><br>All antibodies used were individually titrated before their use to identify their optimal working concentration.<br>All experiment included appropriate isotype control, and FMO (fluorescence-minus-one) controls.

Eukaryotic cell lines

Policy information about cell lines

Cell line source(s)	Ba/F3 (murine pro-B cell; RCB0805) and OP9 (murine osteoblast; RCB1124) were purchased from the RIKEN cell bank (Tsukuba, Japan).
Authentication	All cell lines used in the study were obtained from RIKEN cell bank. See manufacturers websites for authentication information.
Mycoplasma contamination	All cell lines used in the study were negative for mycoplasma contamination.
Commonly misidentified lines (See ICLAC register)	None of the cell lines used in the study were commonly misidentified lines listed in the ICLAC.

Animals and other organisms

Policy information about studies involving animals; ARRIVE guidelines recommended for reporting animal research

Laboratory animals	C57BL/6 mice (Ly5.2) were purchased from Japan SLC (Shizuoka, Japan) and C57BL/6-Ly5.1 mice were purchased from Sankyo Labo Service (Tsukuba, Japan). A2Kio/J (Vav1-iCre) and Trp53tm1Tyj/J (p53+/-) mice were purchased from The Jackson Laboratory (Bar Harbor, ME). Eight-12 weeks old mice (female) were used for all experiments.
Wild animals	This study did not involve wild animals.
Field-collected samples	This study did not involve samples collected from the field.
Ethics oversight	All experiments were carried out under the guidelines of Osaka University Committee for animal and recombinant DNA experiments and were approved by the Osaka University Institutional Review Board.

Note that full information on the approval of the study protocol must also be provided in the manuscript.

Flow Cytometry

Plots

Confirm that:

☒ The axis labels state the marker and fluorochrome used (e.g. CD4-FITC).

☒ The axis scales are clearly visible. Include numbers along axes only for bottom left plot of group (a 'group' is an analysis of identical markers).

☒ All plots are contour plots with outliers or pseudocolor plots.

☒ A numerical value for number of cells or percentage (with statistics) is provided.

Methodology

Sample preparation	Detailed sample preparation for each experiment is presented in the Methods Section of the manuscript. Briefly, fetal liver and bone marrow were dissected form mice and cells harvested by pipetting. Debris and aggregated cells were removed through a nylon-mesh.
Instrument	Flow cytometry and sorting was performed on a FACS Aria (BD Biosciences)
Software	To collect and sort flow cytometry data the FACS Diva software (BD Biosciences) was used. Analysis of the data was performed using FlowJo V10 (Flow Jo LLC).
Cell population abundance	Abundance of relevant cell population within post-sort fraction and purity of sorted cell population was validated from reanalysis by flow cytometry.

Gating strategy

For all flow cytometry experiments debris was removed by gating on size using SSC-A/FSC-A parameters. Gating strategy from viable cells are shown in Supplementary Figure 8. Dead cells were excluded by propidium iodide (Sigma) staining or analyses using the two-dimensional profile of the forward versus side scatter. An unstained sample was used to gate the negative population.

☒ Tick this box to confirm that a figure exemplifying the gating strategy is provided in the Supplementary Information.

This checklist template is licensed under a Creative Commons Attribution 4.0 International License, which permits use, sharing, adaptation, distribution and reproduction in any medium or format, as long as you give appropriate credit to the original author(s) and the source, provide a link to the Creative Commons license, and indicate if changes were made. The images or other third party material in this article are included in the article's Creative Commons license, unless indicated otherwise in a credit line to the material. If material is not included in the article's Creative Commons license and your intended use is not permitted by statutory regulation or exceeds the permitted use, you will need to obtain permission directly from the copyright holder. To view a copy of this license, visit http://creativecommons.org/licenses/by/4.0/
